# Retraction Note: Population-level counterfactual trend modelling to examine the relationship between smoking prevalence and e-cigarette use among US adults

**DOI:** 10.1186/s12889-023-16800-7

**Published:** 2023-10-02

**Authors:** Floe Foxon, Arielle Selya, Joe Gitchell, Saul Shiffman

**Affiliations:** PinneyAssociates Inc, 201 North Craig Street, Suite 320, Pittsburgh, PA 15213 USA

**Retraction Note: BMC Public Health (2022) 22:1940**

10.1186/s12889-022-14341-z

The Editors have retracted this article after readers raised concerns around the methodology. A post-publication review highlighted concerns about the assumption of ‘0’ prevalence of e-cigarette use in 2010 which contradicts available data; this assumption is not fully supported by the sensitivity analyses performed by the authors. Therefore, the Editors have lost confidence in the results reported by the article. None of the authors agree to this retraction.

